# Perceptions and Expectations of Psychology Students Regarding the Qualifications of Undergaduate Psychology Education: A Qualitative Study

**DOI:** 10.1192/j.eurpsy.2026.11525

**Published:** 2026-07-31

**Authors:** S. Yurduşen, I. Gözel, B. Kardaş

**Affiliations:** Psychology, Ankara Science University, Ankara, Türkiye

## Abstract

**Introduction:**

The quality and adequacy of undergraduate psychology education are central to shaping students’ professional identity and career plans. Yet little is known about how students evaluate their education while still enrolled. Exploring their perceptions, concerns, and needs may help identify areas for improvement, particularly regarding their sense of competence in applied fields during the early years after graduation.

**Objectives:**

This study aimed to examine psychology students’ perceptions of the quality and adequacy of their undergraduate education in preparing them for professional life, and to identify the areas they believe require improvement.

**Methods:**

The study was conducted with 56 third- and fourth-year psychology undergraduates at Ankara Bilim University. Semi-structured interviews lasting 20–30 minutes were conducted with seven open-ended questions about the adequacy and effectiveness of their training. All interviews were transcribed verbatim and analyzed thematically using NVivo (15th edition) through both inductive and deductive approaches. Each author conducted separate analyses, which were then reviewed collectively at intervals of 5–10 interviews, until consensus on the themes was reached.

**Results:**

The preliminary results revealed 5 main themes: *the strong need for practical experiences and supervision, the high and uni-dimensional workload of theoretical courses, the need for guiding relationships between students and professors*, *anxiety about building a professional identity*, and *anxiety about the lack of legislative requirements in Turkey.* Overall, many students perceived psychology education in Turkey as shallow and overly theory-oriented, insufficient for fostering a professional identity grounded in practical competencies. They emphasized the importance of relational experiences with professors as mentors, the necessity of more practice-oriented courses, and the requirement for postgraduate training. Theoretical courses were frequently described as abstract when detached from applied practice, leading students to perceive themselves more as intellectuals than professionals. Moreover, students expressed the need for clearer legislative definitions to guide their career paths, noting that the lack of concrete boundaries regarding their role in mental health settings generates considerable anxiety.

**Conclusions:**

The findings suggest undergraduate psychology students view their training as insufficient for professional identity formation, while acknowledging its role in personal development. Expanding applied coursework, bridging theory and practice, and clarifying postgraduate pathways may better align education with students’ expectations and needs. The results also underscore the need for clear role definitions, standardized requirements, and intervention policies to strengthen psychologists’ qualifications.

**Disclosure of Interest:**

None Declared

